# Promotion of the excited electron transfer over Ni- and Co -sulfide co-doped g-C_3_N_4_ photocatalyst (g-C_3_N_4_/Ni_x_Co_1−x_S_2_) for hydrogen Production under visible light irradiation

**DOI:** 10.1038/s41598-017-08163-y

**Published:** 2017-08-09

**Authors:** Kai Fan, Zhiliang Jin, Hao Yang, Duanduan Liu, Hongyan Hu, Yingpu Bi

**Affiliations:** 1 School of Chemistry and Chemical Engineering, North Minzu University, Yinchuan, 750021 P.R. China; 2 State Key Laboratory for Oxo Synthesis and Selective Oxidation, Lanzhou Institute of Chemical Physics, Chinese Academy of Science, Lanzhou, 730000 P.R. China

## Abstract

A Ni- and Co- sulfide co-doped g-C_3_N_4_ photocatalyst (g-C_3_N_4_/Ni_x_Co_1−x_S_2_) was prepared by hydrothermal method and this photocatalyst, namely, g-C_3_N_4_/Ni_x_Co_1−x_S_2_ shown excellent photocatalytic properties due to the special structure of Ni-Co-S with boundary different exposure to active site of transition metal-metal (Ni-Co) active planes. With the introduction of Co atoms, the H_2_ production amount reached the maximum about 400.81 μmol under continuous visible light irradiation for 4 hours based on the efficiently charge separation and greatly improved electron transfer resulted from the presence of sufficient active exposure at the boundary. The serial studies shown that the existence of Ni-Co-S structure over g-C_3_N_4_ active surface is the key factor of activity affections by means of several characterizations such as SEM, XRD, XPS diffuse reflectance etc. and the results of which were in good agreement with each other. A possible reaction mechanism over eosin Y-sensitized g-C_3_N_4_/Ni_x_Co_1−x_S_2_ photocatalyst under visible light irradiation was proposed.

## Introduction

Increasingly environmental pollution and global energy shortage have attracted a lot of attention, so it is a priority to find a new type of energy to solve these problems. Hydrogen energy was considered to be one of the mainstream energy in the future, due to it is a kind of clean, low-cost, and environmentally friendly energy^1^. However, according to the reported so many semiconductor photocatalysts which be used to spilt water to product hydrogen^2–4^, according to the report, it is disappointing that low utilization of solar energy can only reach 4% for those catalysis. For practical application requirement this utilization is far cannot be satisfied. So we have to find a new material which possesses high-efficiency, high utilization and more faster for the preparation of hydrogen evolution. Research had shown that by using of semiconductor photocatalysts under visible light to water splitting could improve hydrogen production rate greatly. Among them, TiO_2_ as a photocatalyst had been used under ultraviolet (UV) light to water splitting at first time in 1972, but because of the low hydrogen production efficiency, low utilization of solar energy and high electron hole recombination rate limited its development potential^5^. Like graphitic carbon nitride (g-C_3_N_4_), a metal-free polymeric which possessrs visible light active, energy conservation and environmental, had been reported in 2009 by Wang *et al*. which had attracted the interest of the researchers Yin *et al*. propose a straightforward method to create quantum superposition states of a living microorganism by putting a small cryopreserved bacterium on top of an electromechanical oscillator^6–8^.

It was found that g-C_3_N_4_ exhibits high surface and an appealing electronic band structure, high physicochemical stability, and “earth-abundant” nature^9–13^. And different precursors like urea, thiourea melamine and so forth could transform to g-C_3_N_4_ by heating directly. In short, the production method of g-C_3_N_4_ was very simply, but it has outstanding performance. The other reasons choosed g-C_3_N_4_ as catalyst due to the structures of g-C_3_N_4_ is different from other ordinary photocatalyst. According to the literature, we come to understand that g-C_3_N_4_ with a band gap of approximately 2.7 eV, because of the presence of sp^2^-hybridized carbon and nitrogen, the eatablishment of the π-conjugated electronic structures and the likeness to lamellar structure of carbon materials. Guo L.J and Shen S.H *et al*. reported polymer heterojunction (PHJ) photocatalysts consisting of polyfluorene family poly mers and graphitic carbon nitride (g-C_3_N_4_) for efficient SHC^14–19^. These excellent properties make g-C_3_N_4_ as a heterogeneous catalyst and a semiconductor photocatalyst under visible light irradiation to split water^20, 21^. Nevertheless, it cannot obtain good effect if only made pure g-C_3_N_4_ as a main catalyst. The poor separation efficiency of photogenerated electron-holes leads to limit the efficiency of hydrogen production, but this can be promoted by means of chemical methods, such as design the construction of metal/semiconductor/lsotype/carbon/conducting polymer/sensitizer/multicomponent-g-C_3_N_4_ heterjunctions^5^. There are many kinds of modified methods, and the choice of co-catalyst is a key factor. There is no denying that Pt, Au, Ag and Pd^22^ as co-catalysts have a good effect, because the precious metals for proton reducibility has high activity, while the potential for development is finitude due to the reserves are too rare^20^. Therefore looking for a cheap co-catalyst is the main research direction of the future. The content of the transition metal Ni is believed to be rich, Hong *et al*. used nickel acetate and thioacetamide as raw material, making NiS_2_ deposition on the g-C_3_N_4_ through the hydrothermal method^23^. Lu *et al*. used the similar approach made NiS_2_ deposition on the ultrathin e-C_3_N_4_ nanosheets, it was proved that the NiS_2_ of doping could improve the photocatalytic reaction performance greatly and high stability for hydrogen production under visible light^24^. It can be seen through infrared spectrum that after doping NiS_2_ on the g-C_3_N_4_, the edge was not red-shifted. Zhang *et al*. synthesized cobalt hydroxide/oxide on graphitic carbon nitride whose morphology was stacked and formed floriform. After experiment it was found that slight amount of Co could improve the activity of photocatalytic OER activities like positive effect on electron-hole separation, charge carriers transfer rate and electronic conductivity of g-C_3_N_4_^25^. Although now about g-C_3_N_4_ modification method is very effective, but exploring a new method to improve the efficiency of hydrogen production is still necessary.

The formation of the specific structure promotes the electron-hole separation and charge transportation during the process of the hydrogen production. Compared with precious metals, doped transition metal Ni could make hydrogen production efficiency increases, and Ni is a kind of high content of elements. The g-C_3_N_4_/NiS_2_ was prepared through an ion-exchange method at room temperature. Under the optimal conditions, the hydrogen production rate can achieved 44.77 mol/h, which is similar to the photocatalytic of Pt/g-C_3_N_4_ (2.0 wt% Pt/g-C_3_N_4_) and through the different characterization methods such as photocatalytic hydrogen production, photoluminescence (PL) analysis, and photoelectrochemical (PEC), it is indicating that when the NiS_2_ after doped the separation of photogenerated charge carriers was improved and hydrogen production activity had been greatly improved. However, the yield of hydrogen is limited^13^. Thus, the Ni is a kind of promising metal material.

Recently, the transition metals has attracted widely attention, Liu *et al*. have found the compound of the Co-g-C_3_N_4_@rGO, it delivers great oxygen reduction reaction activity and kinetics, as well as better stability^26^; Zhu *et al*. have synthesized a noble-metal-free photocatalytic system by modifying g-C_3_N_4_ with CoS using impregnation-sulfidation. The conclusion is that after doped the CoS, photocatalytic activity of g-C_3_N_4_ was strongly influenced^27^. Hence the transition metal—Co is a kind of catalyst which could be used to enhance the photoelectric response of materials.

In this work we used cobalt and nickel respectively to decorate carbon nitride graphene (g-C_3_N_4_) by hydrothermal method in order to prepare g-C_3_N_4_/Ni_x_Co_1−x_S_2_ composite photocatalyst. In the process of reaction that was joined Eosin Y, we find that hydrogen production quantity has been improved significantly, the reason may be that due to the existence structure of Ni-Co active surface, which own a large number of different active sites, inhibiting the recombination of electron-hole and the improvement of electron transfer rate. For this purpose, we found that through the contrast experiment the addition of Co could further promote photocatalytic activity. Similarly, the result of fluorescence spectrum is consistent with speculation. And through the experimental data, the peak of Ni-Co-S structure is the lowest, indicating that the charge separation efficiency is higher than others. The effects of the Co content on hydrogen generation were also be investigated. This report may provide new high performance and low energy consumption catalysts for hydrogen generation.

## Result and Discussion

### XRD analysis

**Figure 1:**
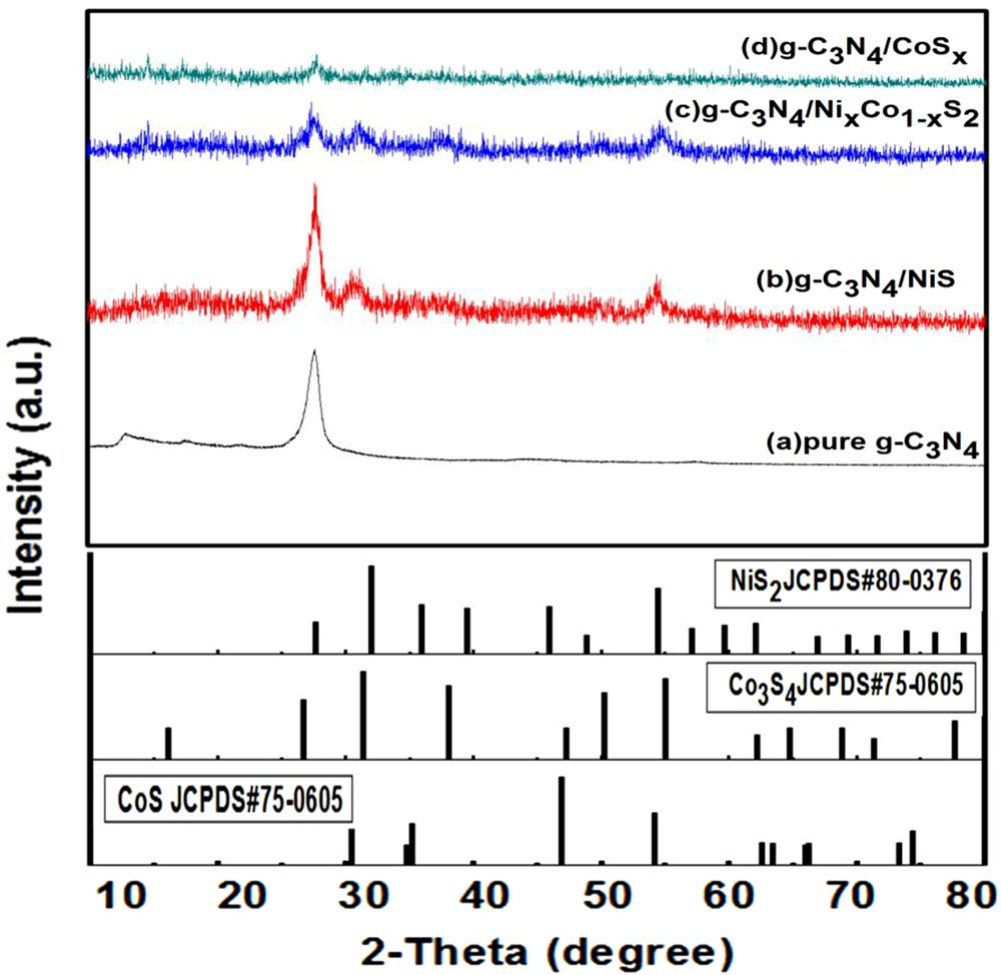
X-ray diffraction (XRD) patterns of pure g-C_3_N_4_, g-C_3_N_4_/NiS_2_, g-C_3_N_4_/CoS_x_ and g-C_3_N_4_/Ni_x_Co_1−x_S_2_ and the JCPDS of NiS_2_, Co_3_S_4_ and CoS.

Through the X-ray diffraction (XRD) patterns we could know whether the g-C_3_N_4_/Ni_x_Co_1−x_S_2_ nanocomposite was synthesized successfully. Figure 1 has shown the patterns of pure g-C_3_N_4_, g-C_3_N_4_/NiS_2_, g-C_3_N_4_/CoS_x_ and g-C_3_N_4_/Ni_x_Co_1−x_S_2_ composite. Figure 1(a) is pure g-C_3_N_4_ patterns which shows two typical peaks. The strongly diffraction peak at 27.35° could be assigned the (002) interplanar and the distance is 0.326 nm, and the other weak peak at 13.1° corresponds to the (100), which is similar to the in-plane structure of tri-s-triazine units of 0.672 nm. By means of XRD, to be sure that the pure g-C_3_N_4_ sample has hexagonal phase, and also be consistent with the JCPDS#87–1526^28^. The diffraction peak of pure NiS_2_ could be indexed for JCPDS 80–0376,which own the main two diffraction peaks at 32.0°, and 54.4° were ascribed to be the (200), and (311) diffraction signals. When the NiS_2_ was doped in g-C_3_N_4_, the g-C_3_N_4_/NiS_2_ sample shown three particular diffraction peaks, and one of the most to the right of the peak is belong to g-C_3_N_4_, other two peaks are ascribed to be NiS_2_^13^, which is similar to the standard cards.Due to the CoS_x_ in this nanocomposite is a compound which maybe contains of cubic Co_3_S_4_ and hexagonal CoS, also the diffraction peaks could be indexed for JCPDS #42–1448 and JCPDS #75–0605 respectively. According to the JCPDS file, the pure Co_3_S_4_ has three discernable diffraction peaks at 26.6°, 31.3° and 55.0° were ascribed to be the (220), (311) and (440) diffraction signals. And the pure CoS has two peaks at 30.6° and 47.1° were attributed to (100) and (102) diffraction signals. Nevertheless compared with pattern of g-C_3_N_4_/CoS_x_, when the Co was loaded g-C_3_N_4_/NiS_2_, the pattern of g-C_3_N_4_/Ni_x_Co_1−x_S_2_ is similar with the pattern of g-C_3_N_4_/NiS_2_. Similarly, the peak of cobalt species could not be found from the pattern. There is a reason of this phenomenon, maybe the low doped percentages of cobalt and small particle was dispersed on the catalyst^29, 30^.Through the XPS could be evidenced the cobalt was doped on g-C_3_N_4_/NiS_2_ successfully.

### SEM characterization

**Figure 2:**
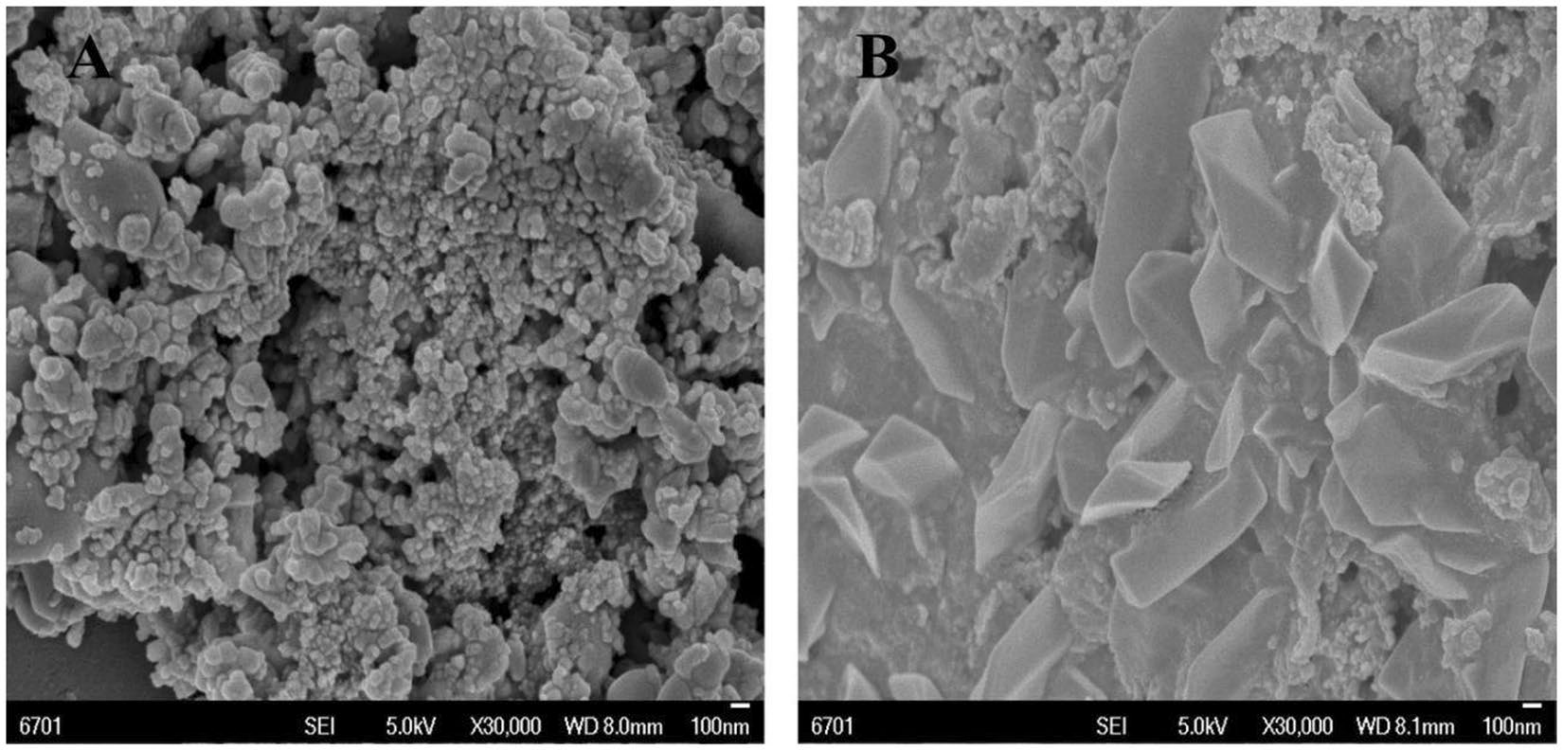
SEM patterns of pure A g-C_3_N_4_ and B g-C_3_N_4_/Ni_x_Co_1−x_S_2_ nanocomposite samples.

The photocatalyst of g-C_3_N_4_/Ni_x_Co_1−x_S_2_ nanocomposite could be analyzed by SEM. An SEM image of pure g-C_3_N_4_ and g-C_3_N_4_/Ni_x_Co_1−x_S_2_ nanocomposite samples is shown in Fig. 2. It can be seen that the structure of pure g-C_3_N_4_ is similar to that of carbon, except that pure g-C_3_N_4_ has a mesoporous structure with π-conjugated structure. Also on the pure g-C_3_N_4_ surface, there are a large number of observable hollow structures (Fig. 2A). In the g-C_3_N_4_/Ni_x_Co_1−x_S_2_ composite catalyst (Fig. 2B) it can be observed that the morphology of the g-C_3_N_4_/Ni_x_Co_1−x_S_2_ composite catalyst is irregularly intertwined with g-C_3_N_4_.

### TEM characterization

**Figure 3:**
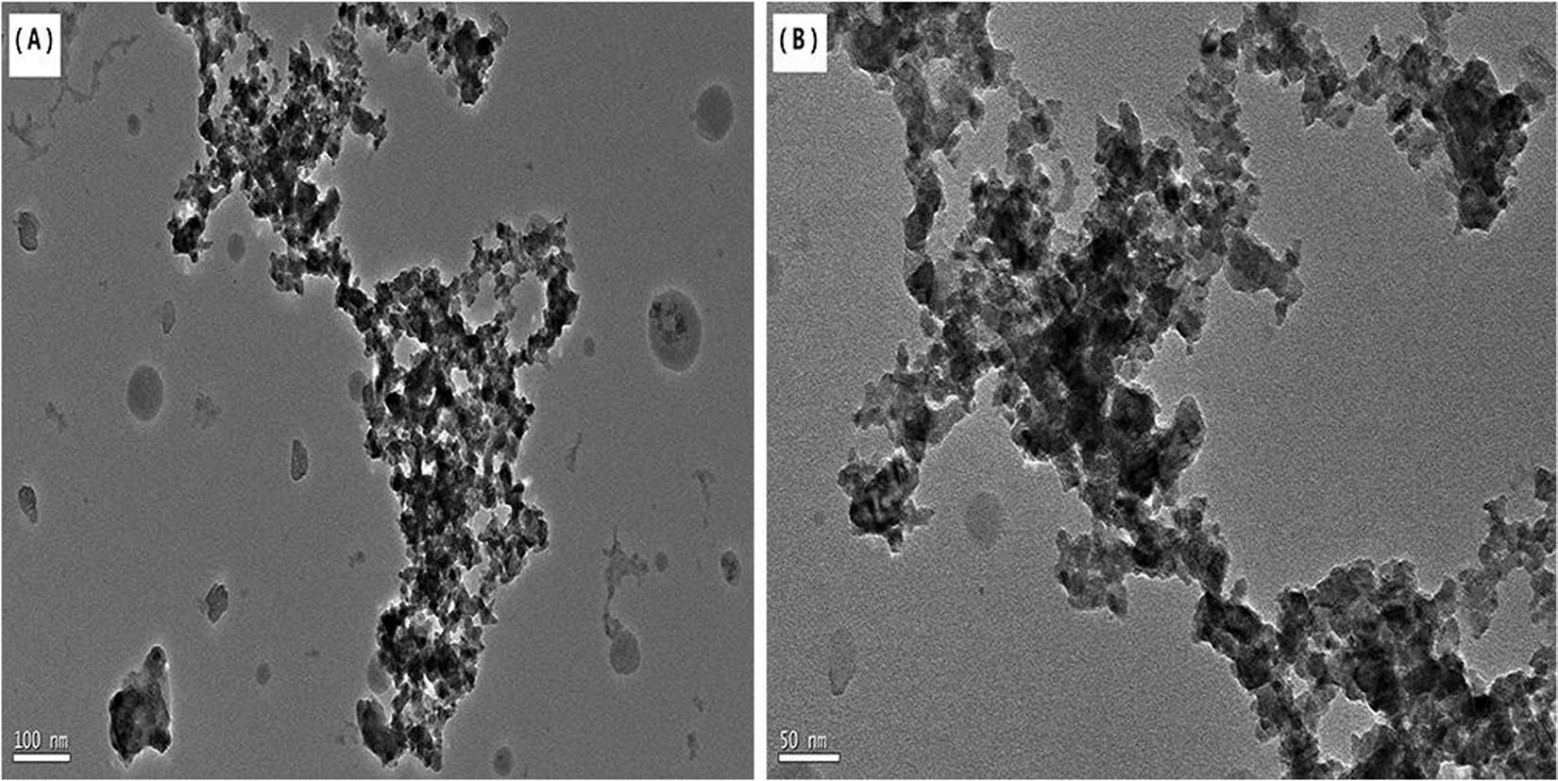
The TEM images of the samples.

Through the Transmission Electron Microscopy (TEM) images, we could observed subtle structural characteristic of the g-C_3_N_4_/Ni_x_Co_1−x_S_2_ nanocomposite clearly. Figure 3(A) shows the low-magnification of the g-C_3_N_4_/Ni_x_Co_1−x_S_2_, not only the g-C_3_N_4_ and the Ni_x_Co_1−x_S_2_ sheets are amorphous structure. There are also a large number of Ni_x_Co_1−x_S_2_ sheets on the layer structure of g-C_3_N_4_, and the sheets is firmly bonded to g-C_3_N_4_, indicating that the two types of g-C_3_N_4_ and Ni_x_Co_1−x_S_2_ semiconductors are connected as special charge transfer channel. Likewise, this unusual structure is more efficient for the photoelectron transport and the reduces the recombination efficiency of electron-hole pairs. Furthermore, it can be observed that the g-C_3_N_4_ sheets can limit the growth of the Ni_x_Co_1−x_S_2_ sheets structure. And in the Fig. 3(B), we can know that lamellar structure of the Ni_x_Co_1−x_S_2_ sheets are overlapped together with the g-C_3_N_4_ sheets which formation multilevel interlaced structure. Nevertheless, due to the image are not clear enough to confirm the d values so it is impossible to determine the crystal plane spacing and crystal type of g-C_3_N_4_/Ni_x_Co_1−x_S_2_ nanohybrid, which testifies that the g-C_3_N_4_/Ni_x_Co_1−x_S_2_ was an amorphous material.

### X-ray photoelectron spectroscopy (XPS)

**Figure 4:**
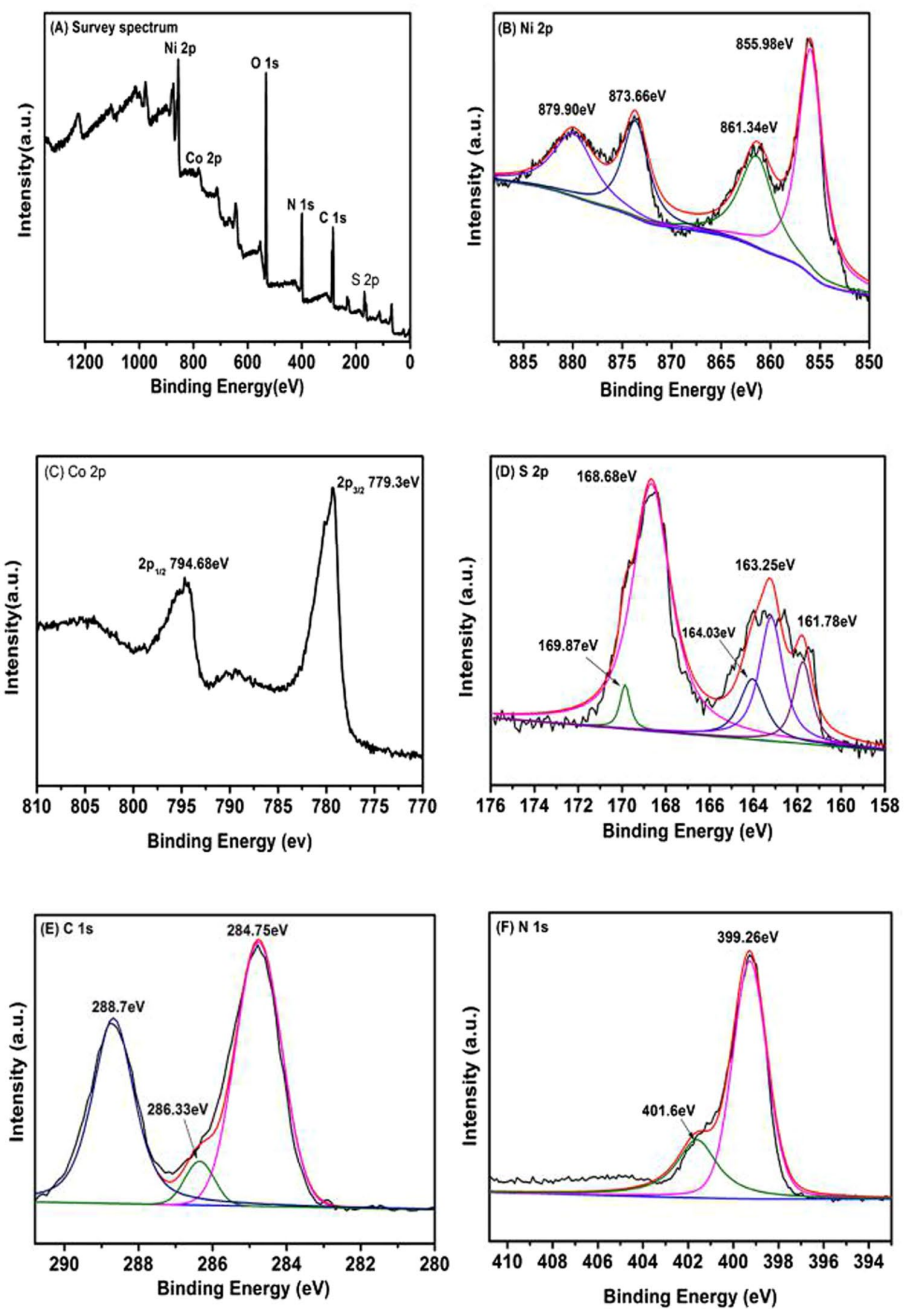
(**A**) XPS survey spectra of the g-C_3_N_4_/Ni_x_Co_1−x_S_2_ sample. (**B**) Ni 2p, (**C**) Co 2p, (**D**) S 2p, (**E**) C 1 s and (**F**) N 1 s scan spectra of the g-C_3_N_4_/Ni_x_Co_1−x_S_2_ sample.

By XPS spectra could clearly understood the information of surface and the characteristics of the composite catalyst element valence state. Figure 4(A) provided the survey spectrum of the composite and showed that the catalyst is composed of Ni, Co, S, C and N elements. As shown in From Fig. 4(B) Ni 2p in the fine spectra could find that it had two characteristic peaks of Ni 2p_1/2_ and 2p_3/2_ respectively, and the corresponding peak was 873.66 eV and 855.98 eV, other two peaks was shake-up satellites, therefore in the g-C_3_N_4_/Ni_x_Co_1−x_S_2_ nanocomposite photocatalyst are characteristic of Ni^2+^ and Ni^3+^ ^31^. In the Co 2p spectrum (Fig. 4C) appeared two characteristic peaks of Co 2p_1/2_ and Co 2p_3/2_ respectively, the position of characteristic peak at 794.68 eV and 779.3 eV, which could be identified in this photocatalyst the Co exists in form of Co^2+^ valence state. Figure 4(D) presents the binding energies of 163.25 eV and 161.78 eV were belong to S 2p_1/2_ and S 2p_3/2_, which was the core-level spectrum of the S 2p. And other peak of S 2p which at around 168.68 eV was ascribed to the SO_4_^2−^ or SO_3_^2−^, the reason of the formation maybe was the S^2−^ of Na_2_S be oxidized in the process of reaction^32^. In the Fig. 4(E), C 1 s presents two single peaks at 284.75 eV and 288.7 eV respectively. The first peak is a specialized standard which show the carbon was the catalyst, however the last peak could be attributed to the carbon atoms bonded with three N atoms in the g-C_3_N_4_ lattice^33^. In Fig. 4F, N 1 s can be deconvoluted into two peaks, at 399.26 eV, which represent the tertiary N bonded to carbon atoms in the form of N–(C)_3_ or H–N–(C)_2_ and the other weaker peak at 401.6 eV was assigned quaternary N bonded three carbon atoms in the aromatic cycles^34^. According to the XPS results, on surface of the nanocomposite contained Ni, Co, and also dopant g-C_3_N_4_ photocatalyst successfully.

### Photocatalytic activities over Eosin Y-sensitized g-C_3_N_4_/Ni_x_Co_1−x_S_2_ for H_2_ evolution

**Figure 5:**
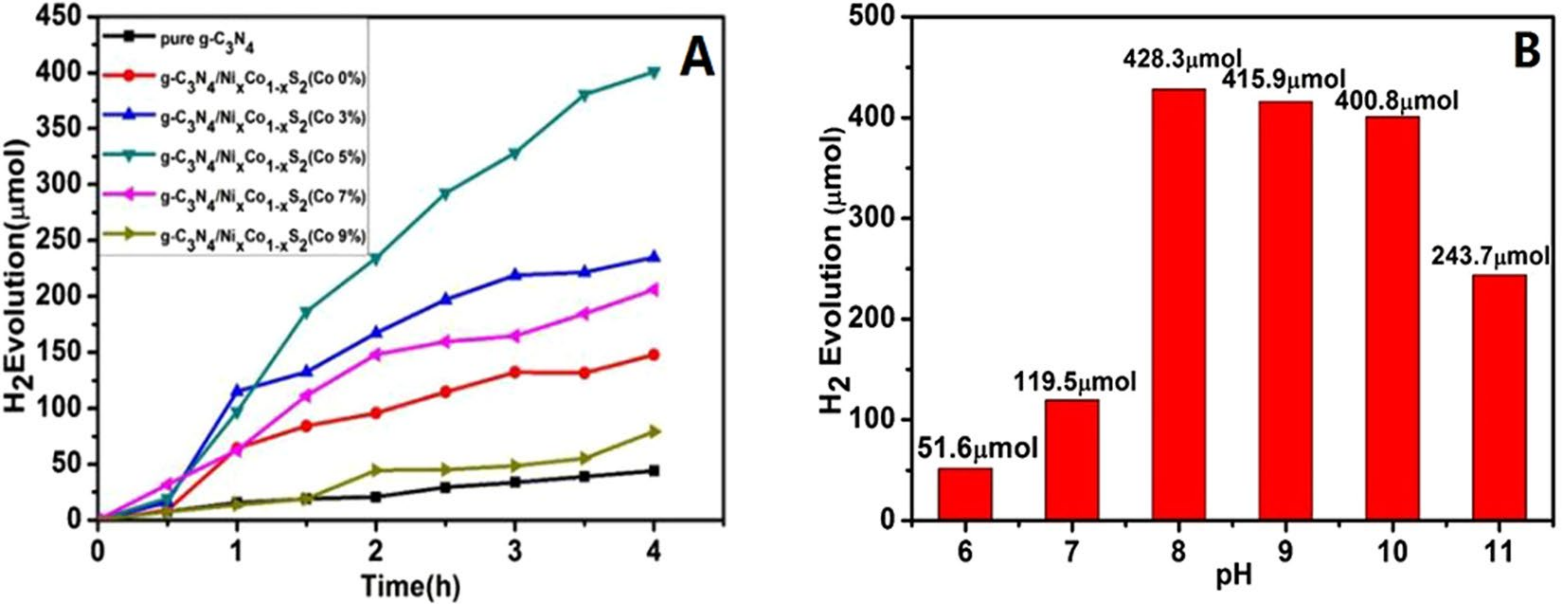
(**A)** Hydrogen evolution over Eosin Y-sensitized catalyst (10 mg) in 15% TEOA aqueous solution (pH = 10) and (**B**). The influence of pH on photocatalytic activity over the g-C_3_N_4_/Ni_x_Co_1−x_S_2_ (Co content 5% and reaction time: 4 h).

Figure 5A shows hydrogen production performance curve of Eosin Y-sensitized pure g-C_3_N_4_, g-C_3_N_4_/Ni_x_Co_1−x_S_2_ (Co content were 0%, 3%, 5%, 7%, 8%, 9%) nanocomposites under visible light (λ ≥ 420 nm). In 100 ml of 15% TEOA (pH = 10) aqueous solution we could clearly know that the production hydrogen capacity of pure g-C_3_N_4_ under visible light irradiation after four hours is only 44.12 μmol, which could clearly manifest the capacity of pure g-C_3_N_4_ to produce hydrogen is the lowest. The reason is that the separation efficiency of its own electron-hole pairs are low exceedingly, leading to the number of electron that escape from its internal to the surface are few. Finally, it will make the probability of electron combined with H^+^ decrease in the catalyst surface, so the amount of H_2_ is less. We doped of Ni elements on the pure g-C_3_N_4_ in order to get a kind of chemical compounds g-C_3_N_4_/NiS_2_. Given the same experimental conditions like light irradiation and the reagent, the hydrogen production after 4 hours could reach 147.77 μmol, indicating the catalytic activity of hydrogen production ability have got improved compared with pure g-C_3_N_4_. We added Co elements during the preparation process of the catalyst, receving g-C_3_N_4_/Ni_x_Co_1−x_S_2_ nanocomposites. The results show that the hydrogen production activity of catalyst had been significantly improved, and when the Co content reached 3%, 7%, 8%, 9%, corresponding to the amount of hydrogen production were 234.88 μmol, 206.00 μmol, 189.21 μmol, and 79.21 μmol. For the same conditions, when Co content reached 5%, the capacity of H_2_ production had reached the maximum value 400.81 μmol, which was 9.08 times than pure g-C_3_N_4_ and 2.71 times than g-C_3_N_4_/NiS_2_ catalysts, respectively. The experimental results suggesting that the cobalt (Co) is a key factor to improve the activity of produce hydrogen, it can also indirectly prove the existence of g-C_3_N_4_/Ni_x_Co_1−x_S_2_ system.

From Fig. 5(B), we could seen that different pH values have a great influence on catalytic hydrogen production in this catalytic reaction system. The catalyst activity of g-C_3_N_4_/Ni_x_Co_1−x_S_2_ photocatalysts become negative extremely in weak acid or strong base environment, contributing to the production of hydrogen has been greatly suppressed. However, in the reaction solution of TEOA as sacrifice electronic agent as well as pH was 8 to 10, the photocatalytic hydrogen production activity was much better. When the pH of solution was 8, the photocatalytic hydrogen production activity reached 428.30 μmol after 4 hours. This phenomenon can be explained as following: 1. In the reaction solution of pH was 8, the special structure of Ni-Co active surface can better exist so that a large number of active site can be more favorable for growth in the edge of transition metal-metal (Ni-Co). 2. Contributing to the favorable growth, the dye molecules could fully effectively adsorbed on the surface of the catalyst, at the same time, increasing the reaction area. 3. The solution of TEOA can restore completely quenched triplet (EY^2−^)^*^, making the reaction efficiency to obtain enhances greatly^33^. When the pH of solution decreased to below 7, the hydrogen production performance of the catalyst inclined gradually, guessing the reason is maybe due to reduced hydroxyl ions makes the stability of Ni-Co structure decline, therefore the electronic transmission was obstructed, resulting in the decrease of hydrogen production amount.

### Photoluminescence (PL) analysis

**Figure 6:**
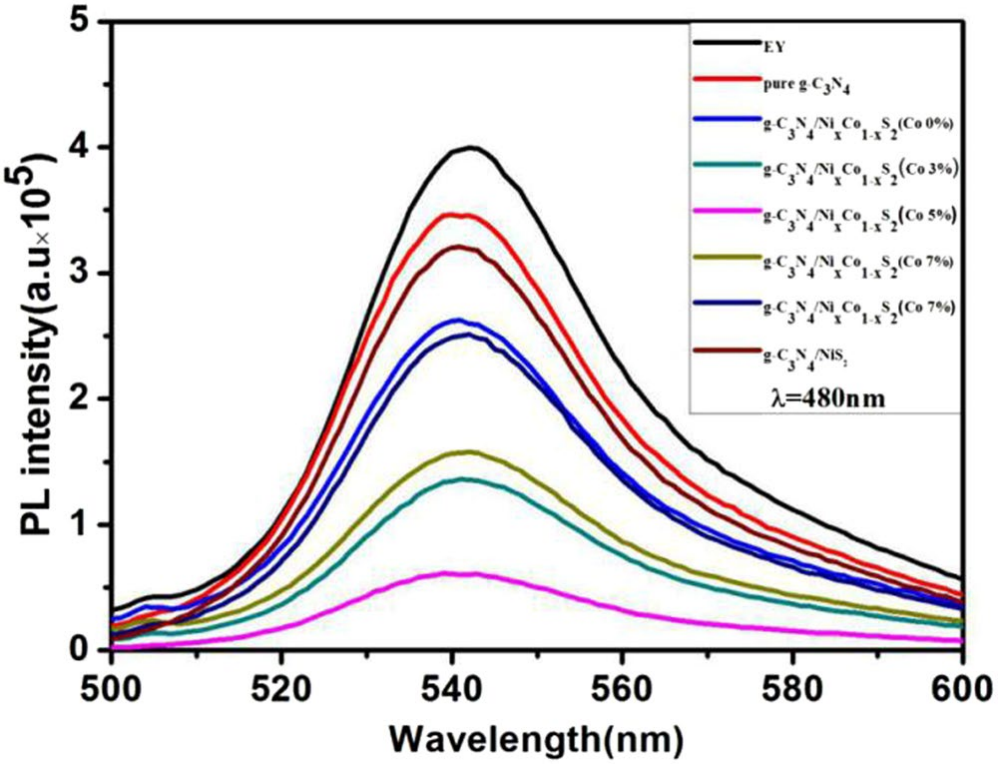
Fluorescence spectra of the g-C_3_N_4_/Ni_x_Co_1−x_S_2_ system.

By steady-state fluorescence spectrum test, we further research the Eosin Y-sensitized g-C_3_N_4_/Ni_x_Co_1−x_S_2_ reaction mechanism (Fig. 6). Under the condition of irradiation, the nanometer compound was triggered, and the ground state electrons will absorb energy to transmit from a low energy level to a higher level, at the same time, the electron-hole pairs were appeared^35^. Then due to the excited electrons were unstable, contributing to they return to ground state right away through the decay of radiative transition process. And the decay of radiative transition process is accompanied by the emission of photons, which produced fluorescence. During this process, the first electronic excitation singlet is returned to the ground state also returned to the electron hole. As shown in Fig. 6, EY-g-C_3_N_4_ produced strongest fluorescence under the excitation of 480 nm light, and its largest emission peak was at 540 nm. When other compounds are sensitized by EY, it can be found that the fluorescence intensity was droped sharply, especially the g-C_3_N_4_/Ni_x_Co_1−x_S_2_ (Co content 5%), reached the peak valley. This kind of phenomenon could have the following explanation: the fluorescence intensity was different which showed that the different compound degree of electron-hole about the different active exposure site in this catalyst system. When the fluorescence intensity is, the peak is higher, showed that the amount of excited electrons returned to ground state which are transited by radiation decay was more, resulting in higher electronic recombination efficiency. On the contrary, the different composites the fluorescence intensity is gradually become low for the different composites, the efficiency of excited electrons transition by radiation decay back to electronic-hole is very poor. The phenomenon is stated that the emission of photons were little and the compound of composite electronic-hole pairs were given a certain inhibition. In this reaction process, we conclude that the photo-generated electron will shift to the conduction band^36^. The reason of the formation is that due to the special Ni-Co-S structure, which have different boundary active sites. It could more fully adsorbed the dye molecules on the catalyst surface, greatly to facilitated the transmission of electrons, improving the charge separation efficiency and photocatalytic hydrogen production activity.

In order to further investigate the interaction between the excited dye EY and the catalyst, the fluorescence lifetime of EY, EY-g-C_3_N_4_, EY-g-C_3_N_4_/NiS_2_, EY-g-C_3_N_4_/CoS_x_, and EY-g-C_3_N_4_/Ni_x_Co_1−x_S_2_ were detected respectively. It can be clearly seen that the fluorescence quenching efficiency was not identical due to different semiconductor catalysts from Table 1. The fluorescence quenching efficiency of EY-g-C_3_N_4_ is the lowest (about 13.3%) among them. When the Ni-Co-S structure was added, the fluorescence quenching rate of EY-g-C_3_N_4_/Ni_x_Co_1−x_S_2_ reached the highest (84.8%). Because of the dye molecules EY and g-C_3_N_4_ have weaker interactions, the complexes formed have weaker fluorescence. The result of lifetime decay following double-exponential indicated the fluorescence quenching of EY-g-C_3_N_4_/NiS_2_, EY-g-C_3_N_4_/CoS_x_, and EY-g-C_3_N_4_/Ni_x_Co_1−x_S_2_ belongs to the dynamic quenching, which indicated that the interaction between EY and complex is strong. In addition, due to the strong interaction force, the more dye molecules can be adsorbed on the surface of composite, and formed two kinds of luminescent bodies. It is well known that the greater transmission efficiency of the photogenerated electrons result the lower recombination efficiency of the electron hole pair, as a result of that, the photocatalytic activity of hydrogen production was improved by the adsorption of more dyes on the catalyst.

**Table 1:** Decay parameters of EY in the presence of g-C_3_N_4_/NiS_2_, g-C_3_N_4_/CoS_x_, and g-C_3_N_4_/Ni_x_Co_1−x_S_2_ in 15% (v/v) TEOA aqueous solution at pH 10.

Systems^a^	Quchencing efficiency (%)	Lifetime, <τ > (ns)	Average lifetime, <τ > (ns)	χ^2^
EY^b^	—	τ = 0.169	τ = 0.169	1.063572
EY-g-C_3_N_4_^b^	13.3	τ = 0.177	τ = 0.177	1.014623
EY-g-C_3_N_4_/CoS_x_^c^	19.7	τ_1_ = 0.187 τ_2_ = 0.1931	τ = 0.1904	1.002758
EY-g-C_3_N_4_/NiS_2_^c^	34.3	τ_1_ = 0.081 τ_2_ = 0.45	τ = 0.19925	1.002713
EY-g-C_3_N_4_/Ni_x_Co_1−x_S_2_^c^	84.8	τ_1_ = 0.223 τ_2_ = 0.216	τ = 0.2198	1.00345

^a^Decay of TEOA aqueous solution (15% v/v) of 1.0 × 10^−6^ mol L^−1^ EY at pH 10 was recorded in the presence of 0.15 mg mL^−1^ EY, EY-g-C_3_N_4_, EY-g-C_3_N_4_/NiS_2_, EY-g-C_3_N_4_/CoS_x_, and EY-g-C_3_N_4_/Ni_x_Co_1−x_S_2_. The excitation and emission wavelengths were 480 nm and 540 nm, respectively. ^b^Single-exponential fit for EY, EY-g-C_3_N_4_, ^c^Double-exponential fit for EY-g-C_3_N_4_/NiS_2_, EY-g-C_3_N_4_/CoS_x_, EY-g-C_3_N_4_/Ni_x_Co_1−x_S_2_.

### Speculation on the mechanism for H_2_ evolution

**Figure 7:**
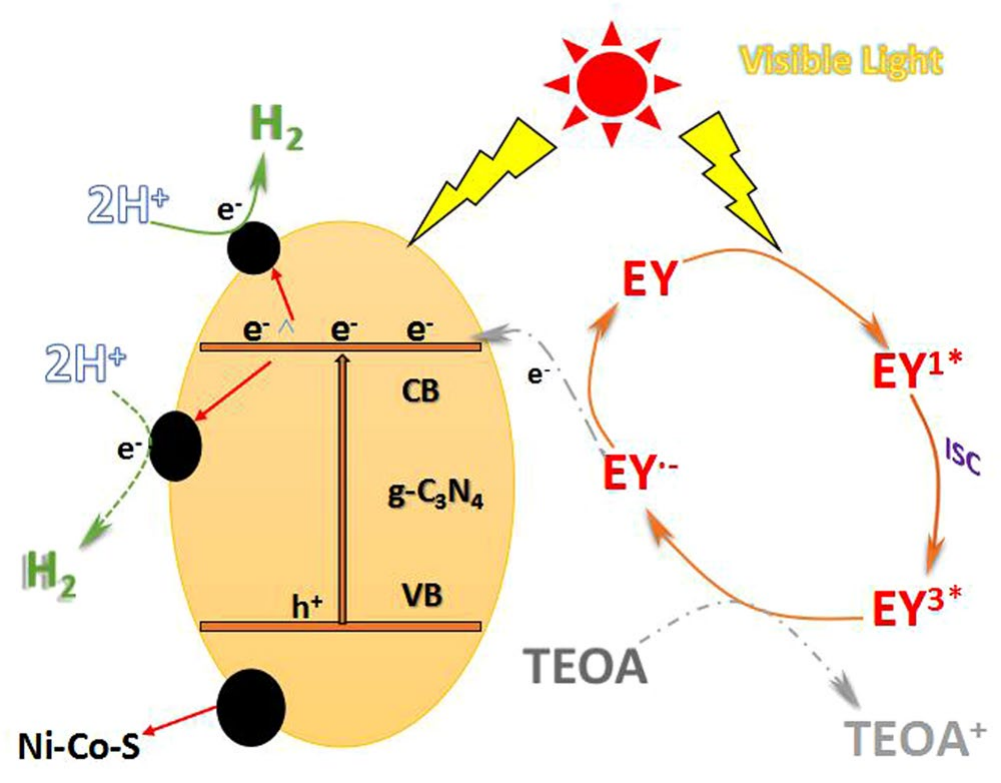
The proposed photocatalytic mechanism for hydrogen evolution over EY-g-C_3_N_4_/Ni_x_Co_1−x_S_2_ photocatalysts under visible light irradiation.

Results from the above discussion, the reaction mechanism of hydrogen evolution over Eosin Y-sensitized g-C_3_N_4_/Ni_x_Co_1−x_S_2_ photocatalysts under visible light irradiation could be explained in Fig. 7. With the addition of transition metal cobalt, the activity of photocatalytic hydrogen production has been greatly improved. The more the active surface of Ni-Co-S structure is more exposed, the more the EY dye molecules can be adsorbed on the surface of the catalyst. These active surface have varying degrees of unsaturation. Simultaneously, these unsaturation will provide more active sites, in order to facilitate the adsorption of dye molecules in the reaction^31^. Under the visible light irradiation, about g-C_3_N_4_, the electrons in the valence band (VB) are excited to the conductive band (CB), and the electrons which were excited would be transferred to g-C_3_N_4_ surface but most excited electrons will reach Ni_x_Co_1−x_S_2_ structure through g-C_3_N_4_. Then the H^+^ in the reaction solution would combine with the excited electrons to form hydrogen, and the electron hole pairs what been left by electron excitation, would react with TEOA in the solution and TEOA would be oxidized become TEOA^+^. Similary, at the reaction aqueous solution the Eosin-Y (EY) was absorbed on the surface of the g-C_3_N_4_ sheets and the Ni_x_Co_1−x_S_2_ sheets in the reaction aqueous solution respectively, which would make the EY molecule jump to the singlet excited state EY^1*^ through absorbing photons. Then the lowest-lying triplet excited state EY^3*^ was generated via an efficient intersystem crossing (ISC). Subsequently the excited state EY^3*^ can be reductively quenched by TEOA to form EY^−^_._ In addition, the TEOA was oxidized to TEOA^+^^37^. And the electrons which belong to EY^−^ were captured by the conduction band (CB) of g-C_3_N_4_ and then transmitted to the Ni_x_Co_1−x_S_2_ directly to product hydrogen. Meanwhile the reduced state dye species get back to the ground state^38^. At present, the process of decomposition of water to hydrogen production has completed. To conclude, the g-C_3_N_4_ after sensitized and doped, could be better as an electronic receiver and transporter, therefore the electron hole recombination efficiency and photocatalytic hydrogen evolution has been significantly improved.

## Conclusions

In summary, a new type of photocatalyst prepared by using transition metal was shown. In this reaction system, the structures of nanocomposite g-C_3_N_4_/Ni_x_Co_1−x_S_2_ and Ni-Co own different boundary exposed active surface and we detected its photocatalytic hydrogen production activity under the visible light irradiation and eosin dye(EY) sensitizion conditions. With the different addition of Co content, the activity of Ni-Co boundary active surface activity has been improved to varying degrees, especially when the Co content reached 5%, the activity of the catalyst reaches the highest value. The synergistic effect between these two different transition metal Ni and Co, which could restrain the recombination of the electron-hole pairs, and improve the electron transfer efficiency. After a series of basic characterization such as XRD, SEM so forth, the catalyst was successfully synthesized, and it is kind of a amorphous material. By the fluorescence spectra(PL) study, we further analyze the separation of photogenerated charge and electron transfer efficiency with the addition of different Co content. And according to the above analysis results, the possible reaction mechanism was proposed. The reason is that contributing to the existence of a special structure Ni-Co with boundary exposure of active surface for this catalyst, the EY dye molecules were fully adsorbed on the catalyst surface, and greatly improve the electronic transmission efficiency. Therefore, under the influence of Ni-Co-S special structure and g-C_3_N_4_/NiS_2_ the photocatalytic activity of nanocomposite g-C_3_N_4_/Ni_x_Co_1−x_S_2_ has been greatly improved. We believe that it is feasible to use transition metal as a metal-free catalyst for the purpose of synthesizing to synthesize a new type of photocatalyst with high performance.

### Experimental section

#### Preparation of g-C_3_N_4_/Ni_x_Co_1−x_S_2_ nanocomposite photocatalysts

The reagent used in the experiment was of analytical grade and was used without any further purification. The preparation of mater-free g-C_3_N_4_: 5 g of melamine was placed on a crucible with crucible cap and directly heated at 550 °C for 4 h in a muffle furnace. The heating reaction was terminated and the muffle furnace was allowed to cool to room temperature, the reaction product was yellow solid and then collect and ground into a powder.

The preparation of g-C_3_N_4_/Ni_x_Co_1−x_S_2_ nanocomposite photocatalysts were used hydrothermal method. Preparations are as follows, 0.125 g of the prepared g-C_3_N_4_ sample was dissolved in 40 mL of ultrapure water and the ultrasonically homogeneously dispersed in an aqueous solution. In order to prepare CoSO_4_•7H_2_O solution at a concentration of 0.0014 mol/L, 0.1 g CoSO_4_•7H_2_O was add to a 250 mL volumetric flask and maintained at a constant volume. Then 0.125 g Ni(NO_3_)_2_•6H_2_O and 6.25 mL of the prepared CoSO_4_•7H_2_O were add to the g-C_3_N_4_ dispersed solution under magnetic stirring. Waiting for all solid have dissolved, we add 25 mL of 0.533 mol/L aqueous Na_2_S•9H_2_O solution to the mixture, and continue stirring. After the mixture was stirred at room temperature for 30 min, the resulting homogeneous solution was transferred to a 100 mL Teflon reaction kettle, heated to 160 °C and kept 10 h. When the reaction time was over, the autoclave was naturally cooled to the room temperature. Finally filtrated to get the reaction production and then washed several times with ultrapure water and ethanol. Dried the production at 40 °C for 5 h. The nanocomposite was fabricated. Also pure g-C_3_N_4_/NiS_2_ was synthesized through the same method.

In accordance with the same method, the g-C_3_N_4_/Ni_x_Co_1−x_S_2_ nanocomposite with different content of Co from 3 to 9% was fabricated.

### Characterization

The X-ray diffraction (XRD) patterns of the samples were recorded using a Rigaku B/Max-RB diffractometer with nickel filtrated Cu Kα radiation operated at 40 kV and 30 mA. Field emission scanning electron microscopy (FESEM) images were recorded using a JSM-6701F scanning electron microscope operated at an accelerating voltage of 5.0 kV. Transmission electron microscopy (TEM) and high-resolution TEM (HRTEM) images were taken using a Tecnai-G2-F30 field emission transmission electron microscope operated at an accelerating voltage of 300 kV. X-ray photoelectron spectroscopy (XPS) measurements were performed using K-Alpha-surface analysis (Thermon Scientific) using X-ray monochromatization. Photoluminescence data (PL) were acquired using a FLUOROMAX-4 spectrophotometer at room temperature.

### Photocatalytic H_2_ evolution experiments

The photocatalytic experiment was carried out by Prefectlight PCX50 A multipass light catalytic reaction system. In this representative photocatalytic experiment, 10 mg of the catalyst was added to 30 mL of a 15% (v/v) triethanolamine (TEOA) aqueous solution. Meanwhile, 20 mg of Eosin Y (EY) dye was added. And then stirred for about 30 min by a magnetic stirrer to allow the EY to be completely adsorbed on the catalyst. The reaction bottle was a cap with a rubber stopper, forming a closed space. The reaction mixture was ventilated with high purity N_2_, with the aim at replacing N_2_ with the gas in the reactants and then followed by continuous light under magnetic stirring condition and measured photocatalytic hydrogen production activity. The amount of hydrogen evolution was measured using gas chromatography (Tianmei GC7900, TCD, 13Xcolumn, N_2_ as carrier).

Before start of the experiment, all glass are was strictly cleaned and washed with ultrapure water.
